# Hypnotic Effects of *Hypericum perforatum* L. and *Melissa officinalis* L. Through Adenosine and Melatonin Receptors

**DOI:** 10.3390/nu18111666

**Published:** 2026-05-22

**Authors:** Hye Jin Jee, Suk Jin Lee, Jae Ryeong Yoo, Hye-Jin Kim, Hyoung-Su Park, Hye-Jeong See, Yi-Sook Jung

**Affiliations:** 1 Department of Pharmacy, Ajou University, Suwon 16499, Republic of Korea; hjjee@ajou.ac.kr (H.J.J.); syudy@ajou.ac.kr (S.J.L.); jryoo@ajou.ac.kr (J.R.Y.); 2 R&D Division, Maeil Health Nutrition Co., Ltd., Pyeongtaek 17714, Republic of Korea; hyejink@maeil.com (H.-J.K.); parkhs@maeil.com (H.-S.P.); hyejeong.see@maeil.com (H.-J.S.); 3 Research Institute of Pharmaceutical Sciences and Technology, Ajou University, Suwon 16499, Republic of Korea

**Keywords:** *Hypericum perforatum* L., *Melissa officinalis* L., adenosine A_1_ receptor, adenosine A_2A_ receptor, melatonin receptor, hypnotic effects, sleep disorder

## Abstract

Background: Sleep disorders, particularly insomnia, represent a major public health concern, while currently available hypnotic drugs are often limited by adverse effects and poor long-term tolerability. Methods: In this study, we investigated the sleep-promoting effects of a mixture of *Hypericum perforatum* L. and *Melissa officinalis* L. extract (HME) and its underlying mechanisms in male ICR and C57BL/6 mice. In a pentobarbital-induced sleep model in mice, sleep onset latency and total sleep time were measured. Pharmacological studies using various antagonists and agonists were conducted to elucidate receptor-mediated mechanisms. Immunohistochemical and immunofluorescence analyses were performed to assess neuronal activity, and cortical mRNA expression was evaluated by quantitative analysis. HPLC analysis was used to identify the major constituents of HME, and their pharmacological profiles were functionally evaluated. Results: HME significantly reduced sleep onset latency and prolonged total sleep time. These hypnotic effects were shown to be mediated through adenosine and melatonin receptor signaling pathways. Immunohistochemical and immunofluorescence analyses showed that HME suppressed neuronal activity in wake-promoting cholinergic and orexinergic neurons of the basal forebrain and lateral hypothalamus, while enhancing activation of sleep-promoting GABAergic neurons in the ventrolateral preoptic nucleus. At the molecular level, HME increased cortical mRNA expression levels of adenosine A_1_ receptor, adenosine A_2A_ receptor, melatonin receptor 1, and melatonin receptor 2. From the HPLC analysis, rosmarinic acid and hyperoside were identified as the major constituents of HME. Functional evaluation of these compounds revealed complementary pharmacological profiles, with hyperoside primarily acting through adenosine receptors and rosmarinic acid engaging both adenosine and melatonin receptor pathways. Conclusions: These findings suggest that HME enhances both sleep initiation and maintenance through adenosine and melatonin receptor signaling pathways, thereby supporting its potential as a multitarget therapeutic agent for improving sleep quality.

## 1. Introduction

Sleep is an essential biological process required for maintaining homeostasis across multiple physiological systems, including metabolic, immunological, and neurocognitive functions [1,2]. Chronic sleep disturbances are increasingly recognized as significant risk factors for a range of health conditions, including cardiovascular disease, diabetes, obesity, depression, and neurodegenerative disorders such as Alzheimer’s disease [3,4]. Insomnia is the most prevalent sleep disorder worldwide. Recent epidemiological projections indicate that approximately 13.9% of the global adult population met the diagnostic criteria for insomnia in 2024, with a significantly higher prevalence observed among women and older individuals [5,6]. These findings underscore the significant public health impact of insomnia and highlight the need for therapeutic strategies with improved safety profiles and well-defined mechanisms of action. Currently, pharmacological treatments for insomnia primarily include benzodiazepines, non-benzodiazepine hypnotics (Z-drugs), and melatonin receptor agonists, which are used to facilitate sleep initiation and prolong sleep duration [7,8]. Although these medications are beneficial in the short term, prolonged use is often associated with adverse outcomes, including tolerance, dependence, cognitive impairment, and disruption of normal sleep architecture [9,10]. These limitations have prompted increasing interest in alternative therapeutic approaches, particularly those based on natural products, which are generally considered to have favorable safety and tolerability profiles.

Sleep regulation is a complex neurobiological process governed by the coordinated interaction of multiple neuromodulatory systems, including γ-aminobutyric acid (GABA), orexin, histamine, serotonin, melatonin, and adenosine [11,12,13]. Given the integrated and interdependent nature of these systems, dysregulation across multiple pathways frequently contributes to sleep disorders such as insomnia [14,15]. Therefore, therapeutic strategies that concurrently modulate several sleep-regulatory pathways are increasingly regarded as more effective than single-target approaches. In this context, plant-derived interventions that can modulate multiple neuromodulatory systems while minimizing adverse effects have attracted growing scientific interest as promising multi-target therapeutic options for the management of insomnia.

Insomnia frequently co-occurs with depressive symptoms, and antidepressant agents have been reported to improve sleep outcomes. Numerous botanicals have been reported to possess sedative and antidepressant properties; however, their sleep-promoting efficacy remains incompletely evaluated [16,17]. In our preliminary study, various natural products with reported antidepressant activities were screened for potential sleep-promoting effects using a pentobarbital-induced sleep model in mice. Mice are widely used animal models in sleep research due to their neurobiological similarities to humans, including cyclic non-rapid eye movement (NREM) and rapid eye movement (REM) sleep patterns, as well as conserved neuromodulatory pathways such as the GABAergic, serotonergic, and melatonergic systems [18,19,20]. Among the tested candidates, *Hypericum perforatum* L. and *Melissa officinalis* L. significantly improved sleep latency and sleep duration. Furthermore, co-administration of these two natural products potentiated the sleep-promoting effects compared with either product administered alone (Figure S1).

*Hypericum perforatum* L. is a medicinal herb traditionally used for the treatment of mild-to-moderate depression and anxiety [21,22]. Its antidepressant efficacy has been well established through numerous clinical studies. A meta-analysis of 23 randomized controlled trials demonstrated that *Hypericum perforatum* L. extracts were significantly superior to placebo and comparable to standard antidepressants, with fewer side effects [21]. Its pharmacological activity is primarily attributed to regulation of the hypothalamic–pituitary–adrenal (HPA) axis and increased expression of brain-derived neurotrophic factor (BDNF) [23,24]. Despite its well-established antidepressant and anxiolytic effects, its sleep-promoting efficacy and underlying molecular mechanisms remain insufficiently explored. *Melissa officinalis* L. has long been used as a beneficial herb due to its antidepressant, antioxidant, anti-inflammatory, and neuroprotective effects [25,26,27,28,29,30]. A randomized double-blind clinical trial demonstrated antidepressant effects comparable to fluoxetine in patients with mild-to-moderate depression [28]. Furthermore, a systematic review and meta-analysis reported significant improvements in depression scores compared with placebo [29]. It is also known to interact with GABAergic signaling pathways, suggesting a potential role in sleep regulation [27,31,32]. In the present study, we investigated whether a combined extract of *Hypericum perforatum* L. and *Melissa officinalis* L. (HME) exerts sleep-promoting effects and, if so, explored the underlying mechanisms responsible for these effects.

## 2. Materials and Methods

### 2.1. Materials

Pentobarbital sodium and diazepam (DZP; positive control) were purchased from Hanlim Pharmaceuticals Co., Ltd. (Seoul, Republic of Korea) and Myungjin Pharmaceuticals Co., Ltd. (Seoul, Republic of Korea), respectively. DPCPX, an adenosine A_1_ receptor (A_1_R) antagonist; SCH58261 (SCH), an adenosine A_2A_ receptor (A_2A_R) antagonist; flumazenil (FLU), a GABA_A_-benzodiazepine receptor antagonist; YNT-185 (YNT), an orexin2 receptor agonist; and luzindole (LUZ), a melatonin receptor antagonist, were purchased from Tocris Biosciences (Avonmouth, Bristol, UK). Primary antibodies included anti-choline acetyltransferase (ChAT; goat), anti-orexin A (OrxA; mouse), and anti-glutamic acid decarboxylase 67 (GAD67; mouse) (Millipore, Burlington, MA, USA); and anti-c-Fos (rabbit) and anti-glyceraldehyde 3-phosphate dehydrogenase (rabbit) (Cell Signaling Technology, Danvers, MA, USA). Secondary antibodies included biotinylated goat anti-rabbit (Vector Laboratories, Burlingame, CA, USA), Alexa Fluor 568-conjugated goat anti-rabbit, and Alexa Fluor 488-conjugated goat anti-mouse (Invitrogen, Carlsbad, CA, USA). Nuclei were counterstained with 4′,6-diamidino-2-phenylindole (DAPI; Molecular Probes, Eugene, OR, USA). HME, as well as the reference compounds (hyperoside, rutin, rosmarinic acid, and caftaric acid), were kindly provided by Maeil Health Nutrition Co., Ltd. (Pyeongtaek, Republic of Korea). All psychoactive substances were used with approval from the Ministry of Food and Drug Safety, Republic of Korea (Approval No. 236).

### 2.2. Preparation of HME and Identification of Active Compounds

The *Hypericum perforatum* L. extract was prepared from dried aerial parts using 60~80% ethanol, followed by filtration, vacuum concentration, and drying. The *Melissa officinalis* L. extract was prepared from dried leaves via hot-water extraction, followed by filtration, vacuum concentration, and drying. The individual extracts of *Hypericum perforatum* L. and *Melissa officinalis* L. (supplier codes: EC841860 and, GA503097, respectively) were supplied by Givaudan France Naturals (Avignon, France). The final complex (4:1, *w*/*w*) of *Hypericum perforatum* L. and *Melissa officinalis* L. extracts was manufactured by Maeil Health Nutrition Co., Ltd. (Gyeonggi, Republic of Korea). This ratio was selected based on preliminary optimization experiments, which demonstrated that the 4:1 combination produced the most pronounced effects on sleep latency and total sleep time at a dose of 100 mg/kg (Figure S2). Quantitative analysis showed that the HME complex contained caftaric acid (0.14 ± 0.01%), rutin (0.63 ± 0.01%), hyperoside (1.02 ± 0.01%) and rosmarinic acid (3.20 ± 0.01%). Phytochemical profiling of the HME was conducted using high-performance liquid chromatography (HPLC) on an Agilent 1260 Infinity II Quat Pump system equipped with a diode array detector (DAD; Agilent, Santa Clara, CA, USA). Separation was achieved on a YMC-Pack Pro C18 column (4.6 × 250 mm, 5 μm). The mobile phase consisted of solvent A (water containing 0.1% trifluoroacetic acid) and solvent B (acetonitrile containing 0.1% trifluoroacetic acid), applied under a multistep linear gradient. The flow rate was maintained at 1.0 mL/min, column temperature at 30 °C, and injection volume at 5 μL. Detection was performed at 325 nm. Based on this analysis, major compounds including caftaric acid, rutin, hyperoside, and rosmarinic acid were identified.

### 2.3. Animals

Male ICR mice (8 weeks old, 25–30 g) and C57BL/6 mice (8 weeks old, 20–25 g) were purchased from Orient Bio (Seongnam, Republic of Korea). All animals were healthy at the start of the study. Both ICR and C57BL/6 mice are wild-type animals and are not genetically modified. No animals underwent any experimental procedures prior to this study. For all animal experiments, the experimental unit was a single animal. ICR mice were used for behavioral and transcriptional analyses due to their widespread use in pharmacological screening, which enables a broader representation of pharmacological responses [33,34]. In contrast, C57BL/6 mice were used for immunostaining owing to their genetic uniformity and reproducibility, which are particularly advantageous for sensitive analyses such as c-Fos immunostaining [35,36]. Approximately 400 male ICR mice were used for the pentobarbital-induced sleep test and quantitative real-time PCR (qRT-PCR). For the immunohistochemistry (IHC) and immunofluorescence (IF) experiments, approximately 50 male C57BL/6 mice were used. All experimental procedures were approved by the Institutional Animal Care and Use Committee (IACUC) of Ajou University (Approval No. 2023-0104) and conducted in accordance with institutional guidelines. All animals were housed at Laboratory Animal Research Center of Ajou University Medical Center under controlled conditions (23 ± 1 °C, 60 ± 10% humidity, 12 h light/dark cycle) with ad libitum food and water, and they were acclimated for at least 5 days before experiments. Animals were monitored daily throughout the study for signs of pain, distress, or abnormal health conditions, including reduced activity, abnormal posture, impaired movement, weight loss, and changes in grooming behavior. Humane endpoints were established in accordance with the institutional IACUC guidelines. Animals showing severe or persistent signs of distress were humanely euthanized by CO_2_ inhalation according to the approved animal care protocol [37]. No adverse events were observed in any experimental group throughout the study period. Group allocation was performed by an investigator who was not involved in outcome assessment, with animals randomly assigned to experimental groups (*n* = 5–10 per group). The investigator responsible for drug administration was aware of the treatment groups, whereas behavioral assessments and data analysis were conducted under blinded conditions. The order of treatments and measurements was kept consistent across all experimental sessions. Animals were randomly assigned to cages to minimize potential cage-location effects. Sample size was determined based on previous studies using similar experimental designs and outcome measures [38,39], and no a priori sample size calculation was performed.

### 2.4. Pentobarbital-Induced Sleep Test

The pentobarbital-induced sleep test, a well-established pharmacological model for evaluating hypnotic activity, sleep latency, and sleep duration, was conducted at 13:00 following a 24 h fasting period. HME, rosmarinic acid, caftaric acid, DZP, and pentobarbital sodium were dissolved in 0.9% saline. Hyperoside and rutin were dissolved in 1% DMSO. Mice in the vehicle control group (CTL) received 0.9% saline, while the experimental groups received HME at doses of 3, 10, 30, or 100 mg/kg or DZP as a positive control at 1 mg/kg. In addition, individual compounds, including hyperoside, rosmarinic acid, rutin, and caftaric acid, were orally (p.o.) administered at doses of 1, 3, 10, or 30 mg/kg. Thirty minutes later, pentobarbital (45 mg/kg, intraperitoneal, i.p.) was administered. FLU (5 mg/kg), DPCPX (5 mg/kg), and SCH (5 mg/kg) were dissolved in 1% dimethyl sulfoxide (DMSO) and administered p.o. 15 min before HME. LUZ (30 mg/kg) and YNT (40 mg/kg) were dissolved in 1% DMSO and administered i.p. 15 min before HME. Sleep onset latency was measured as the interval between pentobarbital injection and loss of the righting reflex, whereas total sleep time was measured as the interval between loss and recovery of the righting reflex. These two parameters were used as primary outcome measures for evaluating the hypnotic effects of HME and determining sample size.

### 2.5. Immunohistochemistry (IHC) and Immunofluorescence (IF)

To investigate the neuronal mechanisms underlying the sleep-promoting effects of HME, c-Fos IHC and IF analyses were performed. c-Fos is a well-established immediate early gene widely used as a marker of neuronal activation. IHC staining was performed in the basal forebrain (BF), lateral hypothalamus (LH), and ventrolateral preoptic nucleus (VLPO). Mice were assigned to the following groups: CTL (0.9% saline, p.o.), HME alone (100 mg/kg), and antagonist pretreatment groups in which DPCPX (5 mg/kg, p.o.), SCH (5 mg/kg, p.o.), or LUZ (30 mg/kg, i.p.) was administered 15 min before HME treatment. One hour later, to minimize animal suffering, the mice were deeply anesthetized with ketamine (2 mg/kg, i.p.; Yuhan Co., Ltd., Seoul, Republic of Korea) and xylazine (0.4 mg/kg, i.p.; Bayer Korea, Seoul, Republic of Korea). Adequate anesthesia was confirmed by the absence of a pedal withdrawal reflex [40], and the mice were then transcardially perfused with saline. The brains were then removed, fixed in 4% paraformaldehyde for 24 h, and cryoprotected in 30% sucrose at 4 °C for 48 h. After cryoprotection, the brains were embedded in Tis-sue-Tek O.C.T. compound and frozen at −80 °C for 48 h. Frozen coronal sections were then prepared at 20 μm using a freezing sliding microtome (Leica SM2400, Leica Microsystems Inc., Wetzlar, Germany) at −20 °C. For c-Fos immunohistochemistry, sections were subjected to antigen retrieval in 10 mM sodium citrate buffer (pH 6.0), treated with 3% H_2_O_2_ to quench endogenous peroxidase activity, and blocked with 5% bovine serum albumin in PBS containing 0.1% Triton X-100 for 1 h. Sections were incubated with rabbit anti-c-Fos antibody (1:500) for 48 h at 4 °C. After washing, sections were incubated with a biotinylated goat anti-rabbit secondary antibody (1:500) for 1 h and visualized using the VECTAS-TATIN^®^ ABC kit (Vector Laboratories, Burlingame, CA, USA) with DAB+ substrate (Dako North America, Carpinteria, CA, USA), and c-Fos-positive cells were quantified using standardized ROI criteria. For IF, sections were incubated with rabbit anti-c-Fos (1:400), goat anti-ChAT (1:500; cholinergic neuronal marker), mouse anti-GAD67 (1:500; GABAergic neuronal marker), or mouse anti-OrxA (1:500; orexinergic neuronal marker) primary antibodies for 48 h at 4 °C. After PBS washing, Alexa Fluor 568–conjugated goat anti-rabbit (1:500) and Alexa Fluor 488-conjugated goat anti-mouse (1:500) secondary antibodies were applied for 1 h at room temperature. Nuclei were counter-stained with DAPI (1:500) for 10 min. Fluorescence images were acquired using a confocal laser-scanning microscope (e.g., LSM 710, Carl Zeiss, Jena, Germany), and co-localization analysis was performed to confirm c-Fos expression in ChAT-positive neurons in the BF, GAD67-positive neurons in the VLPO, and OrxA-positive neurons in the LH region, respectively.

### 2.6. Quantitative Real-Time Polymerase Chain Reaction (qRT-PCR)

To evaluate the effects of HME on the expression of sleep-related receptors, mice were assigned to the following groups: CTL, HME, or one of three antagonist pretreatment groups in which DPCPX, SCH, or LUZ was administered 15 min prior to HME. Cortical tissues were collected 1 h after HME administration. The mRNA expression levels of A_1_R, A_2A_R, MT_1_, and MT_2_ were subsequently quantified by qRT-PCR. The collected tissues were immediately snap-frozen in liquid nitrogen and stored at −80 °C until RNA extraction. Total RNA was extracted using TRIzol reagent (Invitrogen, Carlsbad, CA, USA) according to the manufacturer’s protocol. RNA concentration and purity were assessed using a NanoDrop spectrophotometer (Thermo Fisher Scientific, Waltham, MA, USA). Subsequently, 2 μg of total RNA was reverse-transcribed into complementary DNA (cDNA) using the amfiRivert cDNA Synthesis Platinum Master Mix (GenDEPOT, Katy, TX, USA). qRT-PCR was performed using the amfiRivert qGreen Q-PCR Master Mix (GenDEPOT) under the following conditions: initial denaturation at 95 °C for 3 min, followed by 40 cycles of 95 °C for 5 s, 60 °C for 15 s, and 72 °C for 10 s. Reactions were performed in triplicate. The following primer sequences were used to amplify target genes: A_1_R, Forward: 5′-AGAACCACCTCCACCCTTCT-3′, Reverse: 5′-TACTCTGGGTGGTGGTCACA-3′; A_2A_R, Forward: 5′-CCTACCTGACACCTTCAACG-3′, Reverse: 5′-ACAGGAACACTTCCACACCA-3′; melatonin receptor 1 (MT_1_), Forward: 5′-AGGTCTTCATCTCCCAGCAT-3′, Reverse: 5′-GCAGAGGATGAAGAGGAGGA-3′; melatonin receptor 2 (MT_2_), Forward: 5′-TCACAGACACCATCAGCAGC-3′, Reverse: 5′-GCTTGCAGGATGTTCTTGGT-3′; and β-actin (housekeeping gene), Forward: 5′-CCCAGATCATGTTTGAGACCT-3′, Reverse: 5′-ATGTCACGCACGATTTCCC-3′. Relative gene expression was calculated using the ΔΔCt method with β-actin as the internal control.

### 2.7. Statistical Analysis

Data are expressed as mean ± standard error of the mean (SEM). Statistical analyses were performed using GraphPad Prism version 8.0.2 (GraphPad Software, San Diego, CA, USA). Boxplots were constructed using the median, lower quartile (Q1), upper quartile (Q3), and whiskers extending to 1.5 times the interquartile range (IQR). Outliers were predefined as values below Q1 − 1.5 × IQR or above Q3 + 1.5 × IQR and were excluded from subsequent analyses. Group differences were analyzed using one-way analysis of variance (ANOVA) followed by Tukey’s post hoc test for multiple comparisons. Exact *p*-values are reported where applicable. Effect sizes were calculated as eta-squared (η^2^ = SS_between/SS_total). Statistical significance was defined as *p* < 0.05.

## 3. Results

### 3.1. Hypnotic Effects of HME in a Pentobarbital-Induced Sleep Model

**Figure 1:**
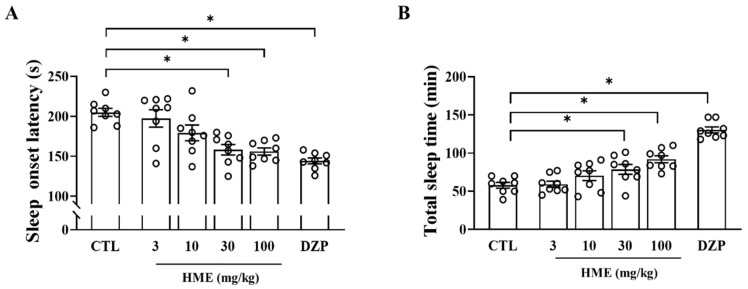
Hypnotic effects of HME in the pentobarbital-induced sleep model in mice. (**A**) Sleep onset latency (one-way ANOVA: F_(5,42)_ = 29.32, *p* < 0.001, η^2^ = 0.571) and (**B**) total sleep time (one-way ANOVA: F_(5,42)_ = 11.20, *p* < 0.001, η^2^ = 0.777) were measured after oral administration of HME (3, 10, 30, or 100 mg/kg) or diazepam (DZP, 1 mg/kg) 30 min prior to pentobarbital injection (45 mg/kg, i.p.). Data are presented as mean ± SEM. A total of 48 mice were used in this experiment (*n* = 8 per group). * *p* < 0.05 vs. CTL. CTL, control; i.p., intraperitoneal; SEM, standard error of the mean.

To evaluate the hypnotic effects of HME, sleep parameters were assessed using a pentobarbital-induced sleep model in mice. Oral administration of HME at doses of 30 and 100 mg/kg significantly reduced sleep onset latency to 158.1 ± 6.602 s and 156.0 ± 4.583 s, respectively, compared with the control (CTL) group (205.0 ± 5.043 s) (Figure 1A). HME at 30 and 100 mg/kg significantly prolonged total sleep time to 78.63 ± 6.434 min and 92.00 ± 4.648 min, respectively, compared with the CTL (57.88 ± 3.815 min) (Figure 1B). DZP (1 mg/kg), used as a positive control, also significantly reduced sleep latency (144.3 ± 3.673 s) and prolonged total sleep time (130.4 ± 3.946 min). Collectively, these results demonstrate that HME exerts dose-dependent hypnotic effects.

### 3.2. Effects of Adenosine and Melatonin Receptor Antagonists on the Hypnotic Effects of HME

**Figure 2:**
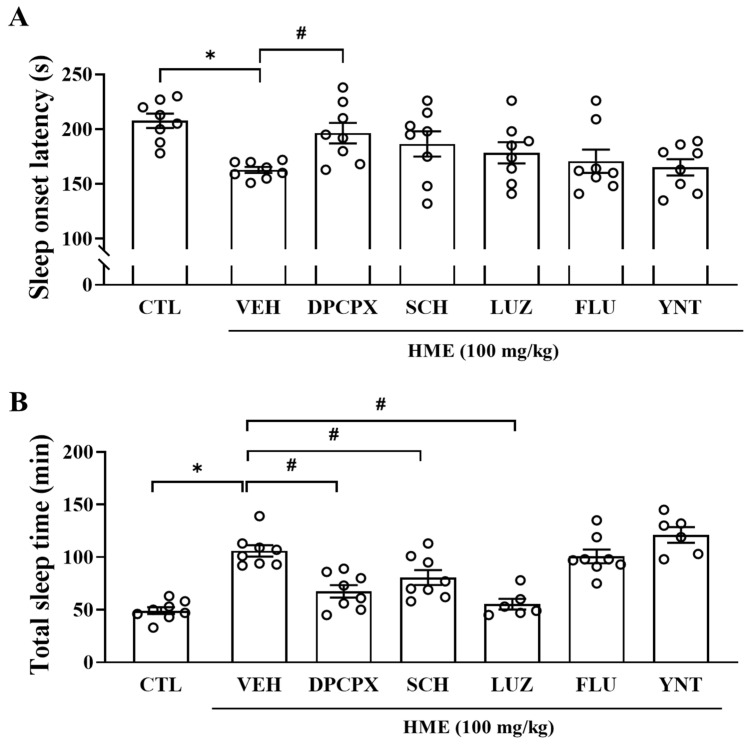
Effects of receptor antagonists and agonist on the hypnotic effects of HME. (**A**) Sleep onset latency (one-way ANOVA: F_(6,49)_ = 3.657, *p* = 0.004, η^2^ = 0.309) and (**B**) total sleep time (one-way ANOVA: F_(6,45)_ = 19.85, *p* < 0.001, η^2^ = 0.726). HME (100 mg/kg, p.o.) was administered 30 min before pentobarbital (45 mg/kg, i.p.), while DPCPX (5 mg/kg, p.o.), SCH58261 (SCH, 5 mg/kg, p.o.), luzindole (LUZ, 30 mg/kg, i.p.), flumazenil (FLU, 5 mg/kg, p.o.), and YNT-185 (YNT, 40 mg/kg, i.p.) were administered 45 min before pentobarbital. Data are presented as mean ± SEM. A total of 56 mice were used in this experiment (*n* = 8 per group). After exclusion of outliers identified by box plot analysis, the final sample size for the total sleep time analysis was *n* = 6 in the LUZ and YNT groups, while the remaining groups retained a sample size of *n* = 8. * *p *< 0.05 vs. CTL; # *p* < 0.05 vs. VEH, VEH, vehicle; p.o., oral administration.

To determine whether the hypnotic effects of HME are mediated via specific sleep-related receptors, the effects of receptor-selective antagonists and agonists were evaluated in the pentobarbital-induced sleep model (Figure 2). In the sleep onset latency analysis (Figure 2A), HME (100 mg/kg) significantly reduced sleep onset latency compared with the CTL (162.8 ± 2.724 s vs. 207.6 ± 6.527 s). This effect of HME was reversed by co-administration of DPCPX, an A_1_R antagonist (196.4 ± 9.398 s). In contrast, co-treatment with SCH, an A_2A_R antagonist; LUZ, a melatonin receptor antagonist; FLU, a GABA_A_ receptor antagonist; or YNT, an orexin-2 receptor agonist, showed no significant effect on the HME-induced reduction in sleep onset latency. In the total sleep time analysis (Figure 2B), HME significantly prolonged total sleep time compared with the CTL (106.0 ± 5.480 min vs. 49.13 ± 3.270 min). The sleep-prolonging effect of HME was significantly attenuated by DPCPX (67.50 ± 5.943 min), SCH (80.63 ± 7.058 min), and LUZ (55.33 ± 5.024 min). However, neither FLU nor YNT affected the HME-induced increase in total sleep time.

Collectively, these findings indicate that HME facilitates sleep initiation primarily through A_1_R activation, whereas A_2A_R and melatonin receptor signaling are involved in its sleep-prolonging effect.

### 3.3. Effects of HME on Neuronal Activity in Sleep–Wake Regulatory Regions in the Mouse Brain

**Figure 3:**
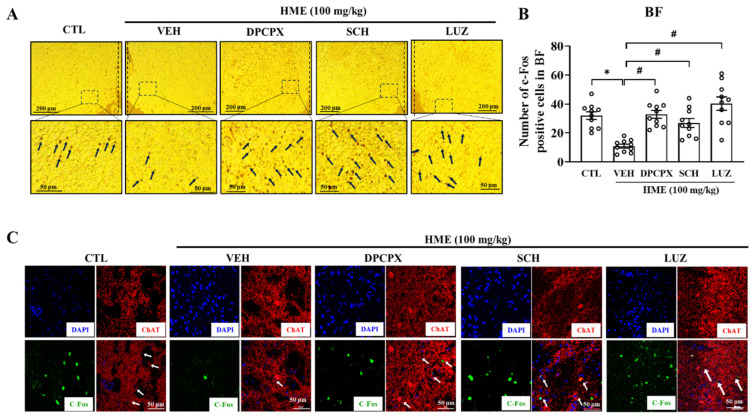
Effects of HME on cholinergic neuronal activity in the BF. (**A**) Representative low- and high-power DAB-stained images of BF regions following HME (100 mg/kg, p.o.) treatment, with or without receptor antagonists: DPCPX (5 mg/kg, p.o.), SCH (5 mg/kg, p.o.), or LUZ (30 mg/kg, i.p.). (**B**) Quantification of c-Fos-positive cells in the BF (one-way ANOVA: F_(4,45)_ = 13.04, *p* < 0.001, η^2^ = 0.537). (**C**) Immunofluorescence images showing ChAT (red), c-Fos (green), and DAPI (blue). Arrows indicate c-Fos-positive cells. Data are presented as mean ± SEM. A total of 50 mice were used in this experiment (*n* = 10 per group). * *p *< 0.05 vs. CTL; # *p *< 0.05 vs. VEH. BF, basal forebrain; ChAT, choline acetyltransferase; DAPI, 4′,6-diamidino-2-phenylindole.

**Figure 4:**
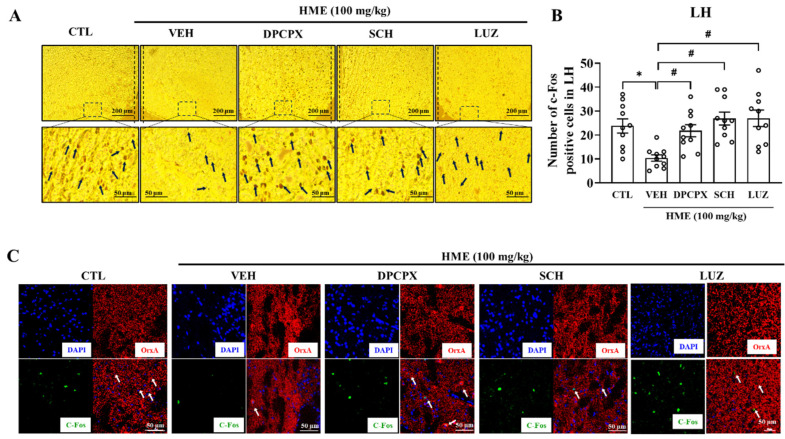
Effects of HME on orexinergic neuronal activity in the LH. (**A**) Representative low- and high-power DAB-stained images of LH regions following HME (100 mg/kg, p.o.) treatment, with or without receptor antagonists: DPCPX (5 mg/kg, p.o.), SCH (5 mg/kg, p.o.), or LUZ (30 mg/kg, i.p.). (**B**) Quantification of c-Fos-positive cells in the LH (one-way ANOVA: F_(4,45)_ = 6.491, *p* < 0.001, η^2^ = 0.366). (**C**) Immunofluorescence images showing orexin-A (OrxA, red), c-Fos (green), and DAPI (blue). Arrows indicate c-Fos-positive cells. Data are presented as mean ± SEM. A total of 50 mice were used in this experiment (*n* = 10 per group). * *p* < 0.05 vs. CTL; # *p* < 0.05 vs. VEH. LH, lateral hypothalamus.

**Figure 5:**
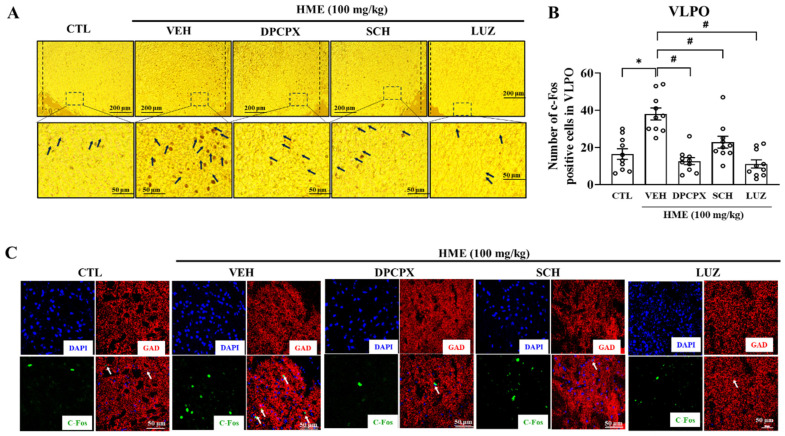
Effects of HME on GABAergic neuronal activity in the VLPO. (**A**) Representative low- and high-power DAB-stained images of VLPO regions following HME (100 mg/kg, p.o.) treatment, with or without receptor antagonists: DPCPX (5 mg/kg, p.o.), SCH (5 mg/kg, p.o.), or LUZ (30 mg/kg, i.p.). (**B**) Quantification of c-Fos-positive neurons in the VLPO (one-way ANOVA: F_(4,45)_ = 16.28, *p* < 0.001, η^2^ = 0.591). (**C**) Immunofluorescence images showing GAD67 (GAD, red), c-Fos (green), and DAPI (blue). Arrows indicate c-Fos-positive cells. Data are presented as mean ± SEM. A total of 50 mice were used in this experiment (*n* = 10 per group). * *p *< 0.05 vs. CTL; # *p* < 0.05 vs. VEH. VLPO, ventrolateral preoptic nucleus; GAD67, glutamate decarboxylase 67.

To investigate whether the hypnotic effect of HME (100 mg/kg) is associated with neuronal modulation in sleep–wake regulatory brain regions, c-Fos IHC and IF analyses were performed in the BF, LH, and VLPO. In the BF (Figure 3A–C), the HME group (VEH) showed a marked reduction in c-Fos-positive cholinergic neurons (10.90 ± 2.637 cells) compared with the CTL (32.00 ± 2.637 cells). This reduction was reversed by the A_1_R antagonist DPCPX (32.90 ± 2.702 cells) and partially reversed by the A_2A_R antagonist SCH (26.90 ± 3.064 cells) and the melatonin receptor antagonist LUZ (40.30 ± 4.568 cells), indicating that HME preserves cholinergic neuronal activity via A_1_R, A_2A_R, and melatonin receptor-mediated pathways. IF analysis further confirmed reduced c-Fos co-localization in ChAT-positive neurons in the HME group (VEH), which was restored by co-administration of antagonists. In the LH (Figure 4A–C), which contains wake-active orexinergic neurons, HME exhibited a significant decrease in c-Fos-positive OrxA neurons (10.40 ± 1.318 cells) compared with the CTL group (23.80 ± 2.969 cells). This reduction was reversed by DPCPX (21.80 ± 2.520 cells) and partially attenuated by SCH (26.90 ± 2.702 cells) and LUZ (27.00 ± 3.432 cells). Consistent with these findings, IF analysis demonstrated reduced c-Fos co-localization in OrxA-positive neurons in the HME group (VEH), which was restored by receptor blockade, suggesting the involvement of adenosine and melatonin signaling in the regulation of orexinergic neuronal activity. In contrast, in the sleep-promoting VLPO (Figure 5A–C), HME significantly increased the number of c-Fos-positive GABAergic neurons (38.00 ± 3.201 cells) compared with the CTL group (16.40 ± 2.802 cells). This HME-induced activation was markedly attenuated by DPCPX (12.60 ± 1.899 cells), SCH (22.80 ± 3.176 cells), and LUZ (11.10 ± 2.228 cells). IF analysis confirmed enhanced c-Fos co-localization in GAD-positive neurons following HME treatment, which was suppressed by antagonists.

Collectively, these results demonstrate that HME modulates neuronal activity within sleep–wake regulatory circuits by suppressing neuronal activity in the BF and LH, while enhancing GABAergic activity in the VLPO. These effects seem to be mediated through A_1_R, A_2A_R, and melatonin receptor signaling.

### 3.4. Effects of HME on Adenosine and Melatonin Receptor mRNA Expression in the Cortex

**Figure 6:**
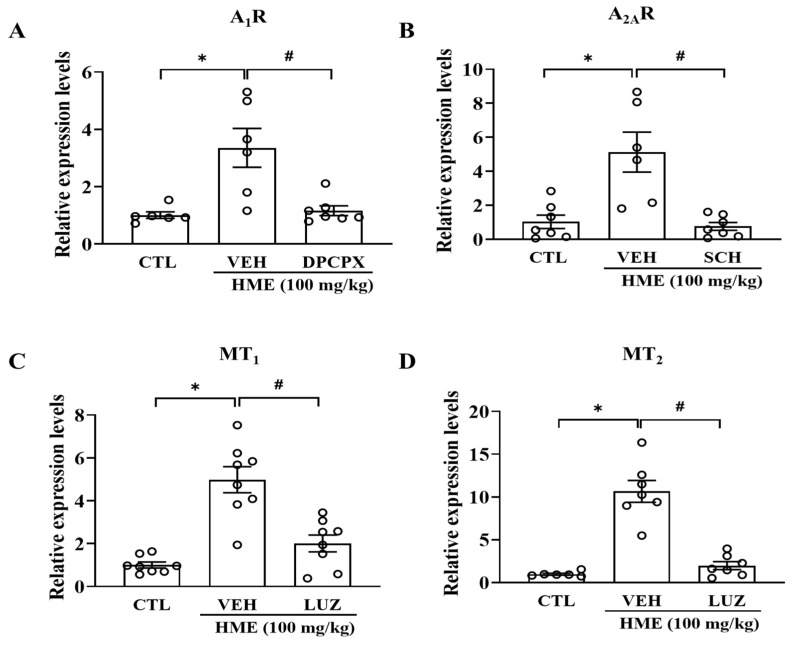
Effects of HME on adenosine and melatonin receptor mRNA expression in the mouse cortex. Mice were administered HME (100 mg/kg, p.o.) 1 h before tissue collection. DPCPX (5 mg/kg, p.o.), SCH (5 mg/kg, p.o.), and LUZ (30 mg/kg, i.p.), were administered 15 min prior to HME. Cortical tissues were harvested for total RNA extraction, and receptor mRNA expression was quantified by qRT-PCR. (**A**) A_1_R (one-way ANOVA: F_(2,15)_ = 9.917, *p* = 0.002, η^2^ = 0.569), (**B**) A_2A_R (one-way ANOVA: F_(2,18)_ = 14.16, *p* < 0.001, η^2^ = 0.611), (**C**) MT_1_ (one-way ANOVA: F_(2,23)_ = 14.81, *p* < 0.001, η^2^ = 0.563), and (**D**) MT_2_ (one-way ANOVA: F_(2,18)_ = 45.32, *p* < 0.001, η^2^ = 0.834) mRNA expression. Data are presented as mean ± SEM. A total of 96 mice were used in this experiment, with eight animals initially assigned to each group (*n* = 8). The final sample sizes used for statistical analysis were as follows: (**A**) CTL (*n* = 6), VEH (*n* = 6), and DPCPX (*n* = 7); (**B**) CTL (*n* = 7), VEH (*n* = 6), and SCH (*n* = 7); (**C**) CTL (*n* = 8), VEH (*n* = 8), and LUZ (*n* = 8); (**D**) CTL (*n* = 6), VEH (*n* = 7), and LUZ (*n* = 7). Reduced sample sizes in some groups resulted from exclusion of outliers identified by box plot analysis. * *p* < 0.05 vs. CTL; # *p* < 0.05 vs. VEH. A_1_R, adenosine A_1_ receptor; A_2A_R, adenosine A_2A_ receptor; MT_1_, melatonin receptor 1; MT_2_, melatonin receptor 2; qRT-PCR, quantitative real-time polymerase chain reaction.

To elucidate the transcriptional regulation underlying the sleep-promoting effects of HME, qRT-PCR analysis was performed on mouse cortical tissues. The mRNA expression levels of adenosine and melatonin receptors were assessed following oral administration of HME (100 mg/kg). As shown in Figure 6, HME significantly increased cortical A_1_R mRNA expression by approximately 3.354 ± 0.678-fold compared with the CTL, and this upregulation was markedly suppressed by co-treatment with DPCPX (1.162 ± 0.171-fold) (Figure 6A). Similarly, A_2A_R mRNA expression was elevated 5.128 ± 1.173-fold following HME administration but was significantly attenuated by SCH (0.667 ± 0.221-fold) (Figure 6B). In parallel, HME administration markedly upregulated melatonin receptor subtypes. The mRNA expression of MT_1_ increased by 4.549 ± 0.691-fold, whereas MT_2_ expression increased by 10.65 ± 1.273-fold relative to the CTL group (Figure 6C,D). Co-treatment with LUZ significantly reversed these effects (MT_1_: 2.163 ± 0.386-fold; MT_2_: 1.820 ± 0.428-fold).

Collectively, these results indicate that HME enhances the expression of adenosine and melatonin receptors at the transcriptional level in the cortex, thereby supporting the involvement of a multi-receptor mechanism underlying its sleep-promoting effects.

### 3.5. HPLC Analysis of HME

**Figure 7:**
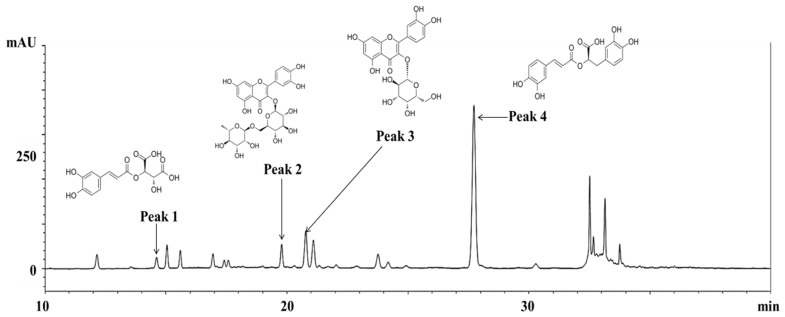
Representative HPLC chromatograms of the HME. The major peaks correspond to caftaric acid (peak 1), rutin (peak 2), hyperoside (peak 3), and rosmarinic acid (peak 4). These compounds were identified by comparing their retention times with those of authentic standards. The corresponding chemical structures are shown in the insets. Detection was performed at 325 nm using a diode array detector wide range. HPLC, high-performance liquid chromatography.

To explore the potential contribution of individual constituents to the sleep-promoting effects of HME, four representative compounds were selected from those identified by liquid chromatography–tandem mass spectrometry (LC-MS/MS) analysis in our preliminary study (Figure S3). As shown in Figure 7, these compounds were confirmed and quantified by HPLC, yielding four peaks corresponding to caftaric acid (peak 1), rutin (peak 2), hyperoside (peak 3), and rosmarinic acid (peak 4). Quantitative analysis revealed that HME contained 1.35 ± 0.01 mg/g of caftaric acid, 6.30 ± 0.04 mg/g of rutin, 10.23 ± 0.06 mg/g of hyperoside, and 31.95 ± 0.04 mg/g of rosmarinic acid.

### 3.6. Hypnotic Effect of HME Compounds in the Pentobarbital-Induced Sleep Model

**Figure 8:**
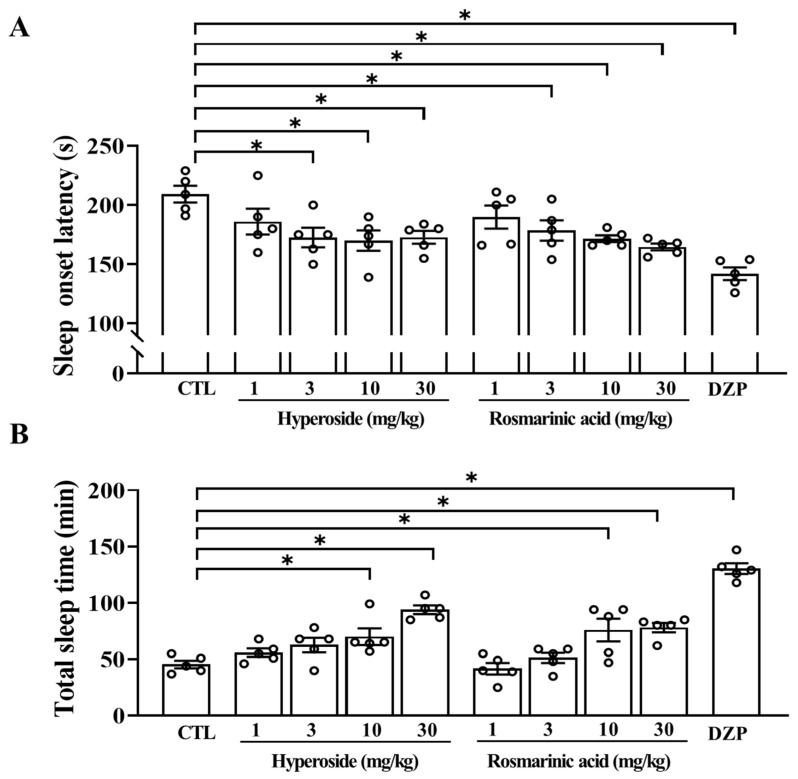
Hypnotic effects of HME-derived compounds in the pentobarbital-induced sleep model. (**A**) Sleep onset latency (one-way ANOVA: F_(9,40)_ = 5.556, *p* < 0.001, η^2^ = 0.556) and (**B**) total sleep time (one-way ANOVA: F_(9,40)_ = 21.74, *p* < 0.001, η^2^ = 0.805) were measured following administration of hyperoside or rosmarinic acid (1, 3, 10, or 30 mg/kg, p.o.) or DZP (1 mg/kg, i.p.), 30 min prior to pentobarbital administration (45 mg/kg, i.p.). Data are presented as mean ± SEM. A total of 50 mice were used in this experiment (*n* = 5 per group). * *p* < 0.05 vs. CTL.

Among the four identified compounds, hyperoside and rosmarinic acid, which were present at relatively higher levels in HME, were selected as representative marker compounds for further evaluation of their sleep-improving effects and underlying mechanisms. The effects of rutin and caftaric acid were also assessed under the same experimental conditions, and these results are presented in the Supplementary Materials (Figure S4). The effects of hyperoside and rosmarinic acid on sleep parameters were evaluated at doses of 1, 3, 10, and 30 mg/kg (Figure 8). In the sleep onset latency analysis (Figure 8A), both hyperoside and rosmarinic acid significantly reduced sleep onset latency at all tested doses compared with the CTL (209.2 ± 7.03 s). Specifically, hyperoside shortened sleep onset latency to 172.6 ± 8.262 s, 170.0 ± 8.620 s, and 172.8 ± 5.380 s at doses of 3, 10, and 30 mg/kg, respectively. Similarly, rosmarinic acid significantly decreased sleep onset latency to 179.6 ± 9.405 s, 171.6 ± 2.874 s, and 164.6 ± 2.891 s at the corresponding doses. The positive control, DZP, reduced sleep onset latency to 142.0 ± 5.339 s. Analysis of total sleep time (Figure 8B) revealed that hyperoside significantly prolonged total sleep time at doses of 10 and 30 mg/kg (70.00 ± 7.403 and 93.80 ± 3.878 min, respectively), whereas rosmarinic acid also significantly increased total sleep time at 10 and 30 mg/kg (75.80 ± 10.08 and 78.00 ± 4.111 min, respectively). As expected, DZP markedly increased total sleep time (130.4 ± 4.760 min). Collectively, these results suggest that hyperoside and rosmarinic acid contribute to the sleep-promoting effects of HME.

### 3.7. Effects of Adenosine and Melatonin Receptor Antagonists on the Hypnotic Effects of Hyperoside and Rosmarinic Acid

**Figure 9:**
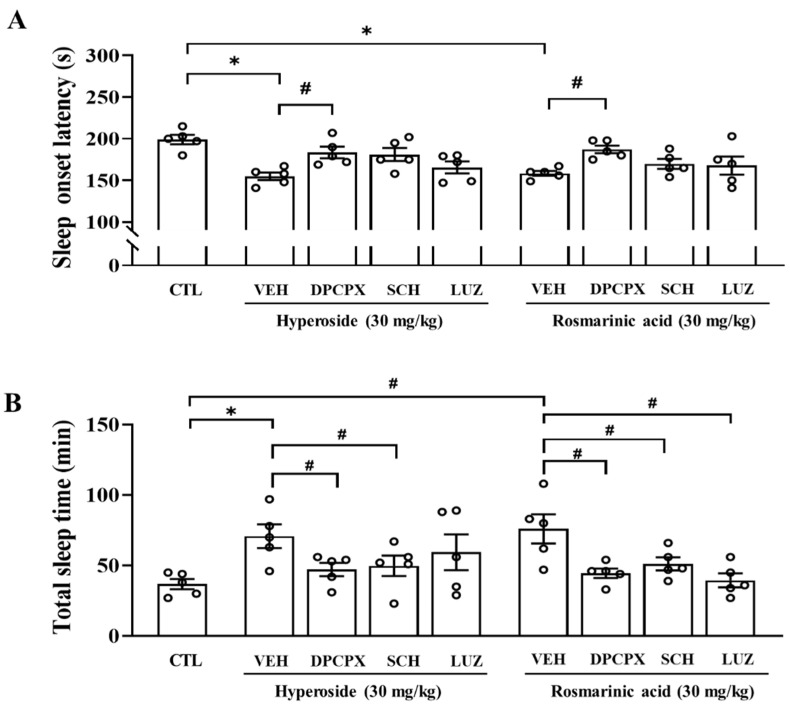
Effects of receptor antagonists on the hypnotic activity of HME-derived compounds in the pentobarbital-induced sleep model. (**A**) Sleep onset latency (one-way ANOVA: F_(8,36)_ = 4.765, *p* < 0.001, η^2^ = 0.514) and (**B**) total sleep time (one-way ANOVA: F_(8,36)_ = 3.361, *p* = 0.006, η^2^ = 0.428) were measured following administration of hyperoside or rosmarinic acid (30 mg/kg, p.o.), given 30 min prior to pentobarbital injection (45 mg/kg, i.p.). DPCPX (5 mg/kg, p.o.), SCH (5 mg/kg, p.o.), or LUZ (30 mg/kg, i.p.) was administered 45 min before pentobarbital. Data are presented as mean ± SEM. A total of 45 mice were used in this experiment (*n* = 5 per group). * *p* < 0.05 vs. CTL, # *p* < 0.05 vs. VEH.

To confirm the receptor subtypes mediating the hypnotic effects of hyperoside and rosmarinic acid, we assessed the effects of selective receptor antagonists on sleep onset latency and total sleep time using the pentobarbital-induced sleep model (Figure 9). Hyperoside (154.8 ± 4.598 s) and rosmarinic acid (158.4 ± 2.943 s) significantly shortened sleep onset latency compared with the CTL (199.0 ± 5.648 s). These effects were restored by co-administration with DPCPX (hyperoside + DPCPX: 183.4 ± 6.918 s; rosmarinic acid + DPCPX: 187.5 ± 5.321 s). In total sleep time analysis, hyperoside significantly prolonged total sleep time (70.08 ± 8.411 min) compared with the CTL group (36.80 ± 3.625 min). This effect was reversed by DPCPX (47.20 ± 4.727 min) and SCH (49.80 ± 7.276 min), but not by LUZ (59.40 ± 12.70 min). Rosmarinic acid significantly increased total sleep time (76.00 ± 10.31 min), and this effect was reversed by DPCPX (44.60 ± 3.370 min), SCH (51.20 ± 4.576 min), and LUZ (39.60 ± 5.006 min).

Collectively, these findings indicate that hyperoside primarily exerts its hypnotic effects through adenosine receptor-mediated pathways, whereas rosmarinic acid acts through multiple receptor pathways involving both adenosine and melatonin receptors.

## 4. Discussion

The present study demonstrates that HME elicits significant hypnotic effects in a pentobarbital-induced sleep model in mice. The HME-induced reduction in sleep latency was blocked only by an A_1_R antagonist, whereas its sleep-prolonging effect was attenuated by antagonists of A_1_R, A_2A_R, and melatonin receptors. HME also decreased neuronal activity in wake-promoting neurons in the BF and LH while increasing the activation of sleep-promoting neurons in the VLPO. In addition, HME upregulated mRNA expression of A_1_R, A_2A_R, MT_1_, and MT_2_ in the cortex. HPLC analysis identified rosmarinic acid and hyperoside as major active constituents. Taken together, these findings indicate that HME elicits sleep-promoting effects through adenosine and melatonin receptor signaling pathways, thereby suggesting its potential as a multitarget therapeutic agent for improving sleep quality.

It has been reported that the activation of A_1_Rs suppresses presynaptic glutamatergic and cholinergic neurotransmission while functionally disinhibiting postsynaptic GABAergic neurons, thereby attenuating arousal and facilitating rapid transition into sleep [38,41]. Accordingly, A_1_R signaling is widely regarded as contributing to both sleep initiation and maintenance [12,42,43]. Consistent with these findings, our results showed that A_1_R blockade by DPCPX abolished the HME-induced reduction in sleep latency and prolongation of sleep duration, indicating that A_1_R activation plays a critical role in the HME-induced promotion of both sleep onset and sleep maintenance. In contrast, the role of A_2A_R in sleep regulation remains controversial, with some studies reporting its involvement in both sleep initiation and maintenance [44,45], whereas others suggest that it plays a preferential role in sleep maintenance [46,47]. A_2A_Rs are enriched in GABAergic neurons of the VLPO and activate the intracellular cyclic adenosine monophosphate (cAMP)–protein kinase A–cAMP response element-binding protein (CREB) signaling pathway, which has been reported to promote sleep-related gene expression [48,49]. The selective A_2A_R agonist CGS21680 increases non-REM and REM sleep duration, enhances delta activity, and activates VLPO neurons [50,51]. Collectively, these findings suggest that A_2A_R is more closely associated with sleep maintenance. Consistent with this notion, the present study demonstrated that blockade of A_2A_R by SCH reversed the HME-induced increase in sleep duration, but not the reduction in sleep latency, indicating a preferential contribution of A_2A_R to HME-induced sleep maintenance. Melatonin is known to regulate sleep through MT_1_ and MT_2_ receptors, which are involved in circadian rhythm regulation and sleep promotion [52,53]. MT_1_ receptors in the suprachiasmatic nucleus and VLPO regulate neuronal excitability during circadian transitions, whereas MT_2_ receptors support sleep stability through extracellular signal-regulated kinase (ERK)-CREB signaling pathways [54,55,56]. Consistent with this physiological role, clinical studies have shown that melatonin primarily increases total sleep duration without significantly altering sleep onset latency [57]. In the present study, blockade of melatonin receptors by LUZ restored the HME-induced increase in sleep duration, but not the decrease in sleep latency, indicating that melatonin signaling mainly contributes to sleep maintenance. Taken together, our findings suggest that the sleep-improving effects of HME may be mediated through distinct yet complementary mechanisms, with A_1_R primarily involved in sleep initiation and A_1_R, A_2A_R, and melatonin receptors contributing to sleep maintenance, although the possibility of off-target effects of the pharmacological antagonists used cannot be entirely excluded.

At the molecular level, HME significantly increased cortical mRNA expression levels of A_1_R, A_2A_R, MT_1_, and MT_2_, supporting the involvement of a multi-receptor mechanism underlying its sleep-promoting effects. These findings further suggest that HME may not only produce acute hypnotic effects but also induce transcriptional changes associated with endogenous sleep regulation. Consistent with previous reports of adenosine receptor upregulation during homeostatic responses to sleep deprivation [58,59,60], the concurrent increase in adenosine and melatonin receptor expression observed in this study supports coordinated regulation of sleep-promoting pathways, which may contribute to sleep stabilization and recovery. However, as mRNA levels do not necessarily reflect functional receptor activity, these findings require further validation. In addition, while cortical adenosine receptor expression has been shown to reflect homeostatic sleep pressure [58,59,61,62], further region-specific analyses targeting sleep-regulatory nuclei such as the VLPO and hypothalamus are needed to better define the underlying neuroanatomical mechanisms. In brain regions that regulate sleep and wakefulness, HME showed significant effects on neuronal activity. Specifically, HME significantly reduced c-Fos expression in wake-promoting cholinergic neurons of the BF and orexinergic neurons of the LH, while enhancing activation of sleep-promoting GABAergic neurons within the VLPO. This coordinated suppression of arousal-promoting networks together with activation of sleep-promoting pathways provides circuit-level support for the behavioral hypnotic effects. These findings indicate that the hypnotic activity of HME involves integrated modulation of multiple nodes within the sleep–wake network rather than effects on a single neuronal population.

HPLC analysis identified rosmarinic acid and hyperoside as the primary bioactive constituents mediating the hypnotic effects of HME. Indeed, rosmarinic acid and hyperoside are widely distributed across diverse plant species, and their anxiolytic and sedative properties have been reported in many preclinical studies [63,64,65,66,67]. Rosmarinic acid has been demonstrated in our previous study to promote sleep through adenosine A_1_R-mediated signaling [63]. Hyperoside has also been previously reported to exhibit sleep-promoting effects in a randomized, double-blind, placebo-controlled clinical trial showing improvements in sleep efficiency and wake after sleep onset [68]; however, the precise receptor-level mechanisms underlying these effects had not been clearly elucidated. In this study, rosmarinic acid was found to exert hypnotic effects through the coordinated modulation of adenosine A_1_R, A_2A_R, and melatonin receptor pathways. These findings extend our previous work by demonstrating that, in addition to A_1_R-mediated signaling, melatonin receptor pathways may also contribute to the sleep-promoting effects of rosmarinic acid, suggesting a more comprehensive multi-receptor mechanism of action. Notably, hyperoside was found to exert hypnotic effects through the coordinated modulation of adenosine A_1_R and A_2A_R signaling pathways, with no apparent involvement of melatonin receptor pathways. These findings provide new mechanistic insight by demonstrating that the sleep-promoting effects of hyperoside are mediated through specific adenosine receptor pathways, thereby contributing to a more detailed understanding of its neuropharmacological actions. Based on the results showing that the doses of individual compounds used in the present study exceeded their estimated exposure levels within the effective HME dose, it is suggested that synergistic interactions among these constituents may enable pharmacological activity at lower concentrations than those required when each compound is administered alone. Consistently, HME exhibited enhanced sleep-promoting effects compared with the individual extracts (Figure S1), supporting the notion that synergistic interactions among constituents derived from the two plants contribute to its hypnotic efficacy. Although rutin and caftaric acid also exhibited sleep-promoting effects (Figure S4), their contributions to the overall hypnotic activity are likely limited due to their relatively low abundance in the extract. However, their potential synergistic roles within the complex mixture cannot be excluded. Collectively, these findings indicate that the hypnotic efficacy of HME arises from coordinated actions of multiple bioactive constituents, supporting a multi-component mechanism of sleep regulation. In addition to receptor-mediated mechanisms, the anti-inflammatory properties of HME constituents may contribute to its sleep-promoting effects. Rosmarinic acid and hyperoside have been reported to suppress neuroinflammatory pathways, including NF-κB-mediated production of pro-inflammatory cytokines such as IL-1β and TNF-α [69,70]. Given the well-established bidirectional relationship between neuroinflammation and sleep disturbance [71,72], this anti-inflammatory activity may represent an additional mechanism underlying the hypnotic effects of HME. Although hypericin and hyperforin have been reported as major contributors to the psychotropic effects of *Hypericum perforatum* [73,74], neither compound was detected in the HME by LC-MS/MS analysis, likely due to differences in raw material sourcing and extraction conditions. Similarly, while the essential oil constituents of *Melissa officinalis* L., such as citronellal and geranial, have been reported to exert anxiolytic and CNS-modulatory effects [75], these volatile compounds are not effectively extracted by hot-water extraction used in this study [76], and their contribution to the sleep-promoting effects observed in the present study is therefore considered minimal.

## 5. Conclusions

This present study demonstrates that HME enhances both sleep initiation and maintenance through the coordinated modulation of adenosine and melatonin receptor signaling. The findings are consistent with our hypothesis that HME exerts sleep-promoting effects. Given the complex neuropathophysiology of insomnia, the multi-target action of HME highlights its potential as a therapeutic candidate. However, the present study has several limitations that should be considered. Although the pentobarbital-induced sleep model is widely used to evaluate hypnotic activity, it primarily reflects the potentiation of drug-induced sedation rather than physiological sleep processes and does not adequately assess sleep architecture, including REM and NREM sleep stages. Therefore, the observed reductions in sleep latency and increases in total sleep time should be interpreted as indicators of hypnotic activity rather than direct measures of physiological sleep. Moreover, these findings should be interpreted with caution in the context of human sleep physiology, as mice and humans differ substantially in their circadian organization and sleep architecture. Despite these limitations, the receptor-specific pharmacological responses and region-specific neuronal modulation observed in this study suggest that HME may exert sleep-promoting effects through adenosine- and melatonin-related signaling pathways. Further studies using EEG-based polysomnography and clinical investigations are required to clarify the effects of HME on physiological sleep architecture and its translational relevance to humans.

## Data Availability

The data presented in this study are available from the corresponding author upon reasonable request, as they are part of an ongoing government-funded research project.

## Supplementary Materials

The following supporting information can be downloaded at https://www.mdpi.com/article/10.3390/nu18111666/s1. Figure S1. Enhanced hypnotic effects of combined extract of *Hypericum perforatum* and *Melissa officinalis* compared with individual extracts in a pentobarbital-induced sleep model.; Figure S2. Effects of different ratios of *Hypericum perforatum* extract to *Melissa officinalis* extract (1:1, 2:1, 4:1) on sleep parameters in the pentobarbital-induced sleep model.; Figure S3. LC–MS/MS analysis of the major compounds in *Hypericum perforatum* and *Melissa officinalis* extracts. Figure S4. Hypnotic effects of HME-derived compounds in a pentobarbital-induced sleep model.

## Abbreviations

The following abbreviations are used in this manuscript:

A_1_R	Adenosine A_1_ receptor
A_2A_R	Adenosine A_2A_ receptor
MT_1_	Melatonin receptor 1
MT_2_	Melatonin receptor 2
HME	Extract of *Hypericum perforatum* L. and *Melissa officinalis* L.
CTL	Control
VEH	Vehicle
LUZ	Luzindole
YNT	YNT-185
SCH	SCH58261
qRT-PCR	Quantitative real-time polymerase chain reaction
HPLC	High-performance liquid chromatography
DZP	Diazepam
BF	Basal forebrain
LH	Lateral hypothalamus
VLPO	Ventrolateral preoptic nucleus
IHC	Immunohistochemistry
IF	Immunofluorescence
cAMP	cyclic adenosine monophosphate
ERK	Extracellular signal-regulated kinase
CREB	cAMP response element-binding protein
SEM	Standard error of the mean
DMSO	Dimethyl sulfoxide
i.p.	intraperitoneal
p.o.	per os (oral administration)
NREM	non-rapid eye movement
REM	rapid eye movement
