# Supplementary material for: Hypnotic Effects of Hypericum perforatum L. and Melissa officinalis L. Through Adenosine and Melatonin Receptors
Source: Nutrients. 2026 May 22;18(11):1666. doi: 10.3390/nu18111666 (PMC13258432; doi:10.3390/nu18111666)
Supplement: Supplementary file 1 [file nutrients-18-01666-s001.zip › nutrients-4208477-supplementary.pdf]

Supplementary Material

Hypnotic Effects of *Hypericum perforatum* L. and *Melissa officinalis* L. through Adenosine and Melatonin Receptors

Hye Jin Jee [1†], Suk Jin Lee [1†], Jae Ryeong Yoo [1], Hye-Jin Kim [3], Hyoung-Su Park [3], Hye-Jeong See [3] and Yi-Sook Jung [1,2,*]

† These authors contributed equally to this work.

*** Correspondence:**

Yi-Sook Jung: yisjung@ajou.ac.kr

Supplementary Figures

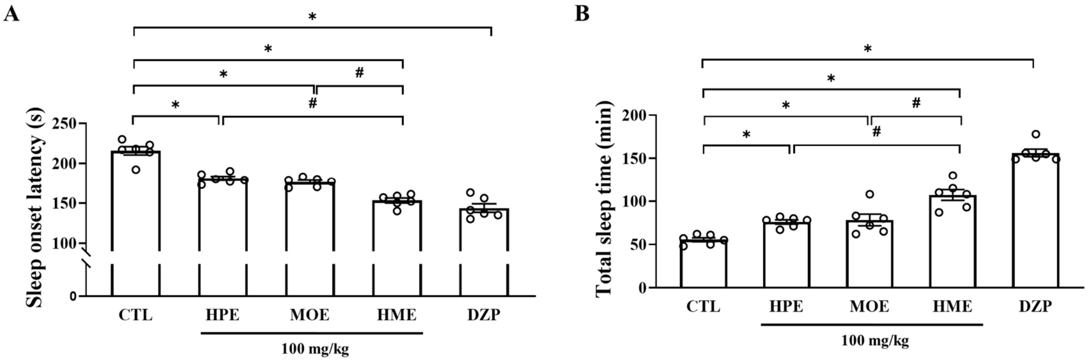

Figure S1. Enhanced hypnotic effects of combined extract of *Hypericum perforatum* and *Melissa officinalis* compared with individual extracts in a pentobarbital-induced sleep model. (A) Sleep onset latency (one-way ANOVA: $F_{(4, 24)} = 48.69$, $p < 0.001$, $\eta^2 = 0.890$) and (B) total sleep duration (one-way ANOVA: $F_{(4, 25)} = 56.04$, $p < 0.001$, $\eta^2 = 0.903$) were evaluated following oral administration of *Hypericum perforatum* extract (HPE), *Melissa officinalis* extract (MOE), or their combined extract (HME) at 100 mg/kg, or diazepam (DZP, 1 mg/kg), 30 min prior to pentobarbital injection (45 mg/kg, i.p.). Values are expressed as the mean ± SEM (n = 6). Statistical significance was determined by one-way ANOVA followed by Tukey's post hoc test. *$p < 0.05$ *vs.* CTL, #$p < 0.05$ *vs.* HME. CTL, control; i.p., intraperitoneal; SEM, standard error of the mean.

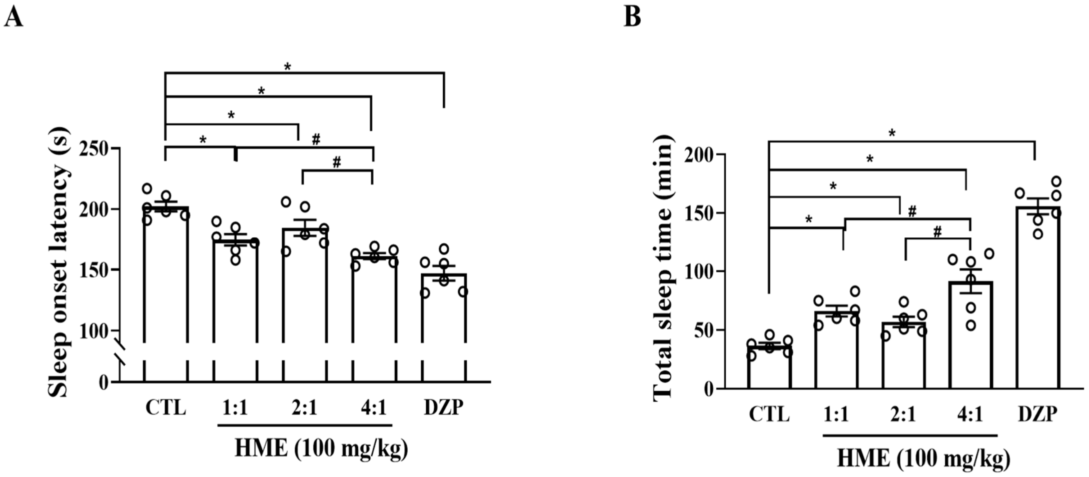

Figure S2. Effects of different ratios of *Hypericum perforatum* extract to *Melissa officinalis* extract (1:1, 2:1, 4:1) on sleep parameters in the pentobarbital-induced sleep model. (A) Sleep onset latency (one-way ANOVA: $F_{(4, 15)} = 17.87$, $p < 0.001$, $\eta^2 = 0.740$) and (B) total sleep time (one-way ANOVA: $F_{(4, 25)} = 53.14$, $p < 0.001$, $\eta^2 = 0.895$) were measured following oral administration of HME at three different ratios of HPE:MOE (1:1, 2:1, and 4:1, w/w) at a fixed total dose of 100 mg/kg, 30 min prior to pentobarbital injection (45 mg/kg, i.p.). Diazepam (DZP, 1 mg/kg, p.o.) was used as a positive control. Data are presented as mean ± SEM (n = 6). Statistical significance was determined by one-way ANOVA followed by Tukey's post hoc test. *$p < 0.05$ *vs*. CTL. #$p < 0.05$ *vs*. 4:1 ratio. CTL, control; HME, combined mixture of Hypericum perforatum and Melissa officinalis extract; DZP, diazepam; SEM, standard error of the mean; i.p., intraperitoneal; p.o., per os.

A

Base peak chromatogram of *Hypericum perforatum* extract (positive mode)

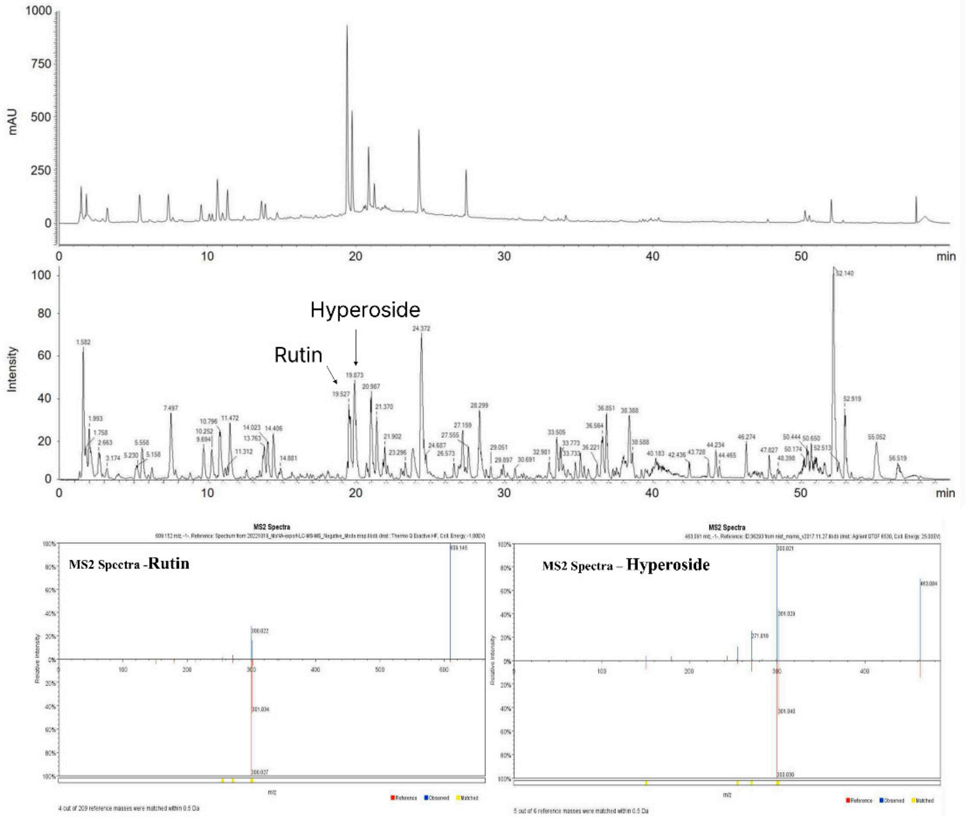

B

Base peak chromatogram of *Melissa officinalis* extract (negative mode)

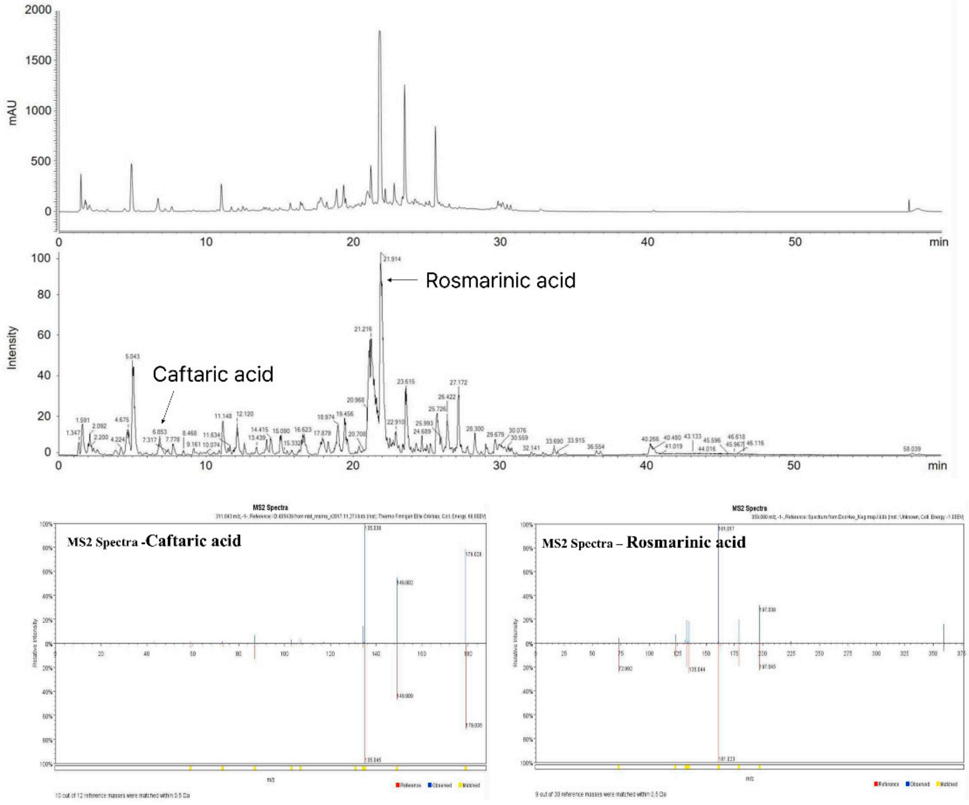

Figure S3. LC–MS/MS analysis of the major compounds in *Hypericum perforatum* and *Melissa officinalis* extracts. (A) Base peak chromatogram of *Hypericum perforatum* extract acquired in positive ionization mode (upper panel) and the MS2-annotated chromatogram indicating the retention times of rutin and hyperoside (middle panel), along with the corresponding MS2 spectra of rutin (lower left panel) and hyperoside (lower right panel). (B) Base peak chromatogram of *Melissa officinalis* extract acquired in negative ionization mode (upper panel) and the MS2-annotated chromatogram indicating the retention times of caftaric acid and rosmarinic acid (middle panel), along with the corresponding MS2 spectra of caftaric acid (lower left panel) and rosmarinic acid (lower right panel). Each compound was identified based on its precursor ion (m/z) and characteristic MS2 fragmentation pattern, and further confirmed by comparison with authentic standards analyzed under identical chromatographic conditions. Blue bars, observed ions; red bars, reference ions from spectral library; yellow bars, matched ions.

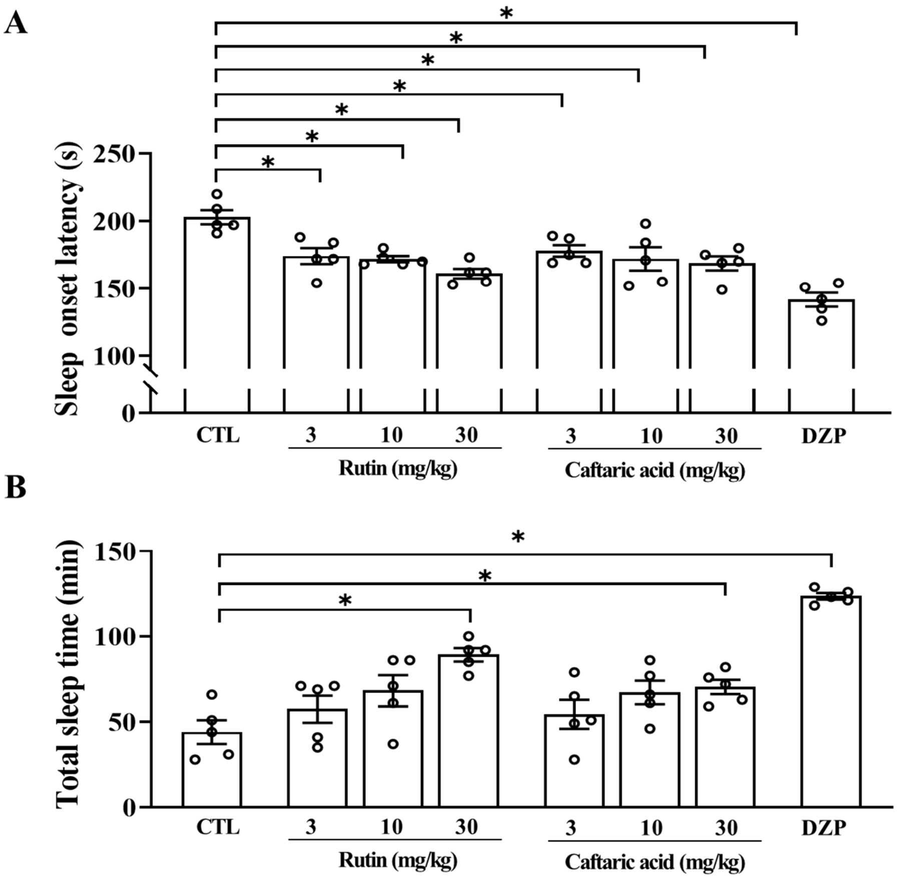

Figure S4. Hypnotic effects of HME-derived compounds in a pentobarbital-induced sleep model. (A) Sleep onset latency (one-way ANOVA: $F_{(7, 32)} = 10.21$, $p < 0.001$, $\eta^2 = 0.691$) and (B) total sleep time (one-way ANOVA: $F_{(7, 32)} = 13.95$, $p < 0.001$, $\eta^2 = 0.753$) were measured following administration of rutin or caftaric acid (3, 10, or 30 mg/kg, p.o.) or DZP (1 mg/kg, i.p.), 30 min prior to pentobarbital administration (45 mg/kg, i.p.). Data are presented as mean ± SEM (n = 5). *$p < 0.05$ *vs*. CTL.
